# Exploring the dual relationship between fintech and financial inclusion in developing countries and their impact on economic growth: Supplement or substitute?

**DOI:** 10.1371/journal.pone.0315174

**Published:** 2024-12-05

**Authors:** Chadi Azmeh, Marwan Al-Raeei

**Affiliations:** 1 Department of Banking and Financial Sciences, International University for Science and Technology, Ghabageb, Daraa, Syrian Arab Republic; 2 Department of Business Administration, Jinan University, Tripoli, Lebanon; 3 Department of Business Administration, University of Kalamoon, Deir Atiyeh, Syrian Arab Republic; 4 Department of Physics, Damascus University, Damascus, Syrian Arab Republic; 5 Department of University Requirements, International University for Science and Technology, Ghabageb, Daraa, Syrian Arab Republic; Bangladesh Institute of Governance and Management, BANGLADESH

## Abstract

This study rigorously examines the complex interplay between financial technology (Fintech), financial inclusion, and their collective effects on economic growth in developing nations. Employing a robust panel regression methodology enhanced by Panel-Corrected Standard Errors (PCSE) and Feasible Generalized Least Squares (FGLS) techniques, this research analyzes an extensive dataset comprising 108 countries categorized into low, lower-middle, and upper-middle income levels across four pivotal years (2011, 2014, 2017, and 2021). Our analysis focuses on two key dimensions of Fintech—specifically, the adoption of digital payments and e-commerce via mobile technologies—and traditional financial access indicators, including the density of ATMs, bank branches, and active banking accounts. The findings demonstrate a predominantly positive effect of Fintech variables on economic growth, particularly through improved digital payment systems. Conversely, traditional financial inclusion metrics frequently show a negative correlation with growth trajectories. Notably, our research underscores a significant positive interaction between digital payment usage and ATM density, indicating a synergistic relationship that enhances the performance of traditional banking systems. In contrast, a substitutability effect arises, where increased dependence on mobile technologies diminishes the relevance of traditional financial infrastructure, potentially obstructing broader economic growth. These findings carry critical policy implications, advocating for a cohesive strategy that fosters both Fintech innovations and traditional financial sectors to maximize economic growth and inclusivity. A deliberate emphasis on synchronizing innovative financial solutions with the strengthening of conventional banking is essential for promoting sustainable economic development in these resource-constrained regions.

## 1. Introduction

Currently, the economic frameworks of developed nations can be characterized as digital economies, symbolizing the continuous technological evolution termed Industry 4.0 [1]. In contrast, developing nations are increasingly focusing on amplifying the influence of Information and Communication Technology (ICT) in their socio-economic growth, alongside the gradual permeation of digital technologies throughout societal structures [2–4]. Modern ICT is recognized as a pivotal driver of economic advancement and efficiency across various sectors by fostering improved automation, optimizing production processes, and enhancing transparency [5–9].

In the financial sphere, the convergence of ICT, innovative technologies, and traditional financial services has triggered substantial shifts within the financial industry. These transformations are encompassed within the larger phenomenon of FinTech innovation, which has reshaped the delivery of financial services and introduced pioneering financial products [10–14]. The Financial Stability Board defines FinTech as “technologically enabled innovation in financial services that could result in new business models, applications, processes, or products with a significant impact on financial markets and institutions, as well as the provision of financial services” [15]. Indeed, FinTech holds the potential to mitigate the shortcomings of conventional microfinance and enhance financial inclusion—ensuring that individuals and businesses can access valuable and affordable financial services tailored to their needs—covering transactions, payments, savings, credit, and insurance—delivered responsibly and sustainably [16]. As such, it has the capacity to improve access to financial services for previously unbanked populations, particularly in developing regions [17–19].

The COVID-19 pandemic has accelerated the adoption of fintech solutions, especially in developing countries, where the demand for remote and easily accessible financial services has surged dramatically. A World Bank study found that mobile money utilization in low- and middle-income nations surged by 65% during the pandemic, highlighting FinTech’s growing importance in ensuring financial inclusion amid escalating social distancing measures [20]. Consequently, the pandemic exacerbated issues such as the diminishing physical banking infrastructure, with certain regions experiencing a nearly 30% reduction in bank branch presence and ATM availability. This shift underscores the urgency of evaluating how fintech technologies might be leveraged to bolster financial inclusion during and after significant global crises [21].

The UN 2030 Agenda emphasizes financial inclusion as essential for realizing Sustainable Development Goals and curbing inequality. Despite some progress, the Global Findex database reveals that 1.7 billion adults still lack access to formal financial services, and an additional 760,000 individuals have access yet do not utilize these services [19, 22, 23]. However, advancements in ICT position FinTech as a promising avenue for enhancing financial inclusion and bridging gaps in bank account ownership and usage. The expanded accessibility of mobile technology provides unparalleled opportunities to harness FinTech to confront these challenges. In particular, mobile financial services are increasingly recognized as a crucial driver of financial inclusion, enabling the underbanked to enter the formal financial system and fostering equitable growth [24, 25].

Previous studies examining the influence of fintech on the economy have primarily concentrated on its effects on various aspects such as financial inclusion [25, 26], poverty alleviation [27, 28], income disparity [23, 29], financial development [30, 31], and overall economic growth [32–34]. However, there has been limited exploration of the potential interplay between fintech and financial inclusion, as well as their reciprocal influence on economic growth. This research seeks to address this oversight by illuminating the underexplored interplay between fintech, financial inclusion, and economic advancement. This inquiry aims to enrich the existing literature and offer insights into how the relationship between fintech innovations and financial inclusion can potentially stimulate economic growth and sustainable progress. Given the pressing need for effective resource allocation in financially constrained settings in developing countries, the study investigates whether fintech innovations and financial inclusion act as complementary forces or substitutes in propelling economic growth. By analyzing the synergistic potential of fintech and financial inclusion, this research aims to provide critical insights for policymakers in developing countries, helping them to refine their resource allocation strategies. Should fintech and financial inclusion be found to support one another, this would highlight the necessity of concurrent investment in both areas to optimize economic benefits. Alternatively, if they are determined to act as substitutes, policymakers could concentrate on fostering one to achieve desirable economic outcomes. Ultimately, this research aspires to enrich the broader dialogue on sustainable development by clarifying how targeted initiatives in fintech and financial inclusion can advance economic growth in resource-limited environments. To accomplish this goal, Panel-Corrected Standard Errors (PCSE) and Feasible Generalized Least Squares (FGLS) methodologies will be applied to analyze data from 108 low, lower-middle, and upper-middle-income countries across four timeframes (2011-2014-2017-2021).

The structure of the paper is organized as follows: Section 2 reviews pertinent empirical literature on FinTech and financial inclusion. Section 3 outlines the data and empirical methodology employed. Section 4 presents the empirical findings, followed by discussion and conclusive remarks in Section 5.

## 2. Literature review

Schumpeter’s seminal publication in 1911 on innovation, particularly regarding finance, has generated substantial interest and engagement from scholars, policymakers, and academics alike. As a result, financial innovation has become an essential factor in driving economic growth and is viewed as a key catalyst for financial advancement in the real economy [35–37]. A considerable volume of empirical studies has repeatedly examined the diverse elements that facilitate financial development, establishing its beneficial effects on economic growth across both advanced and emerging economies [31, 38–45]. Financial innovation encompasses the development of novel and enhanced financial services that significantly boost efficiency in financial transactions, operational processes, and functions within the financial landscape. Early findings indicated that innovations such as ATMs and electronic payments mitigated financial constraints and elevated welfare levels, while later research explored their influence on demand deposits, economic growth, and financial inclusion through mobile technology and fintech [46–50]. Therefore, the nexus between sustainable development and the financial sector is intrinsically linked to financial innovation [51, 52]. Moreover, financial innovation holds the promise of fostering entrepreneurship and job creation, particularly in developing nations where access to conventional banking services is restricted [53, 54].

To delve deeper into the interplay among fintech, financial inclusion, and GDP growth, more refined theoretical models are necessary. Two mechanisms—the financial inclusion channel and the fintech diffusion channel—serve as analytical frameworks for this relationship. The fintech diffusion channel illustrates how the financial services sector can adapt and implement fintech solutions to enhance productivity and efficiency, ultimately elevating living standards [55]. Conversely, the financial inclusion channel emphasizes how fintech can broaden access to financial services, thereby mobilizing savings and steering investments toward impactful objectives within the economy. This framework allows for an examination of the causative relationship and the ways in which these channels interact to influence economic growth [56–58].

### 2.1 Fintech and economic growth

A substantial body of empirical evidence has thoroughly examined the interplay between fintech and its influence on economic growth. Bara et al. [32] conducted a study focusing on the SADC region, revealing a notably positive effect of fintech on economic growth, especially in sectors such as mobile banking and digital payments. Similarly, Qamruzzaman and Jianguo [59] and Qamruzzaman et al. [33] established a robust long-term positive correlation between financial innovation and economic growth in South Asia. Their analysis further indicated that this relationship is nonlinear, suggesting an optimal threshold of financial innovation, beyond which its beneficial effects on economic growth may wane. Additionally, they observed bidirectional causality between these variables in both the short and long term.

Fidan and Guz [60] explored the interplay between GDP and fintech investments across eight high-income countries, uncovering a long-term cointegration between these two factors and a notable cross-sectional dependence. Their findings confirm that fintech investments exert a positive influence on GDP in seven countries, while Singapore exhibited an adverse relationship; short-term Granger causality was detected exclusively in Germany. Moreover, Cevik [34] provided compelling evidence that digital lending acts as a significant catalyst for real GDP per capita growth, particularly in advanced economies. It is crucial to note that fintech has also been shown to enhance competition within the financial sector, resulting in lower costs and improved services for consumers, ultimately leading to economic growth [61, 62].

Furthermore, various country-specific analyses have indicated that fintech exerts a substantial positive influence on real economic growth in China [63–65]. For instance, Song and Appiah-Otoo [66] demonstrated that fintech and its components significantly affect China’s economic expansion, positing that a 10% increase in fintech adoption correlates with an 8% rise in economic growth. Conversely, Mashamba and Gani [67] found that inadequate fintech integration within the financial system has hindered its potential effects on economic growth in Sub-Saharan Africa, noting that while fintech disruptions have led to increased equity funding for banks, they have had a minimal impact on deposit and long-term debt financing. Emerging studies also indicate that fintech can enhance public financial literacy and awareness, fostering a more equitable financial ecosystem—a fundamental requirement for achieving long-term, sustainable economic growth [68, 69]. Parvez et al. [70] concluded that advancements in fintech, coupled with financial inclusion and strong institutional frameworks, positively impact human development in developing Asian nations, suggesting that fintech plays a crucial role in driving inclusive economic growth.

**Hypothesis 1:** The adoption of fintech solutions, particularly in mobile banking and digital payment systems, exerts a positive and significant effect on economic growth in developing countries.

### 2.2 Financial inclusion and economic growth

Numerous empirical investigations have been carried out across global, regional, and national contexts to assess the effect of financial inclusion on economic growth. At the global level, research by Van et al. [71] underscores the positive role of financial inclusion in fostering economic growth. Their study highlights the necessity of increasing access to financial services as a catalyst for entrepreneurship, savings, and investment, ultimately enhancing overall economic prosperity. A stronger relationship emerges in nations with lower income and less financial inclusion. Azimi [72] established a significant bidirectional relationship between financial inclusion and economic growth across different income brackets and regions from 2002 to 2020. While other key determinants, such as private sector credit and foreign direct investment, affect economic growth, they exhibit unidirectional causality without reciprocal influences. Ain et al. [73] investigated financial inclusion’s effects on economic growth specifically within developing nations, revealing a significant and favorable influence. Sethi and Acharya [74] extended this analysis to 31 developed and emerging countries, uncovering a long-term association and bidirectional causation between financial inclusion and economic growth. Kim et al. [75] further examined the Organization of Islamic Cooperation (OIC) countries, identifying a positive impact of financial inclusion on economic growth and confirming reciprocal causality. Fundji [76] established that financial inclusion significantly and positively affects economic growth in both high- and low-income nations, accentuating the critical role of financial inclusion, enhanced by FinTech innovations, in promoting economic development in sub-Saharan Africa. Nonetheless, Sahay et al. [77] found that while financial inclusion initially bolsters economic growth, increased financial development might eventually exert adverse effects on it.

Regional studies consistently affirm the beneficial relationship between financial inclusion and economic growth. For instance, Emara and El Said [78] found a robust positive correlation between financial inclusion and GDP per capita growth in MENA countries with strong institutional frameworks. Likewise, Ifediora et al. [79] focused on sub-Saharan Africa, demonstrating that an increase in bank branches and ATMs significantly benefits economic growth. Additionally, Ali et al. [80] examined IsDB member nations and identified a two-way causation between indicators of financial inclusion and economic growth. Country-specific research has yielded positive associations in India [81], Rwanda [82], and Nigeria [83, 84].

Conversely, a few studies have indicated a negative relationship between financial inclusion and economic growth. Chehayeb and Taher [85] reported an insignificant impact of financial inclusion on economic growth across 13 MENA countries, discovering unidirectional Granger causality from economic growth to financial inclusion, indicating that economic growth enhances both access and usage of financial services. Menyelim et al. [86] found a negative effect of financial inclusion on the relationship between income inequality and economic growth in sub-Saharan African nations. Maune [87] observed adverse effects of financial inclusion and trade openness on economic growth in Zimbabwe, while Nkwede [88] identified a significant negative impact of financial inclusion on economic growth in Nigeria. Lastly, Chiwira [89] provided evidence of a negative long-term relationship between financial inclusion and economic growth within the Southern African Development Community.

The literature highlights important trends regarding the relationship between financial inclusion and economic growth. While the majority of studies affirm a positive correlation, some indicate detrimental impacts, particularly in MENA and African contexts.

**Hypothesis 2:** Financial inclusion significantly and positively influences economic growth, especially in low- and middle-income countries.

The relationship between fintech, financial inclusion, and GDP growth can be explained through two primary mechanisms: the fintech diffusion channel, which enhances productivity and efficiency in financial services, and the financial inclusion channel, which expands access to these services, facilitating savings mobilization and directing investments toward economic growth.

**Hypothesis 3:** The advancement of fintech enhances accessibility to financial services, thereby stimulating economic growth as a complement to financial inclusion.

## 3. Data and methodology

### 3.1 Data

The present research investigates the bidirectional influence of Fintech and financial inclusion on the economic advancement of developing nations. Employing a sophisticated panel regression model that integrates Panel-Corrected Standard Errors (PCSE) and Feasible Generalized Least Squares (FGLS) techniques, this study analyzes a dataset covering 108 countries classified as low, lower-middle, and upper-middle income, across the years 2011, 2014, 2017, and 2021. The selection of these specific years is driven by data availability, as information pertaining to the two key Fintech variables is restricted to these periods, drawn from nationally representative surveys conducted at that time. This analysis incorporates essential variables such as Fintech, indicators of financial inclusion (focused on access and utilization), and a range of factors that contribute to economic growth. Fintech is represented through two specific metrics: the ratio of individuals over the age of 15 who engage in digital payments, and those who conduct e-commerce transactions via mobile devices or the internet. Financial inclusion is assessed using access indicators like the number of ATMs and commercial bank branches per 100,000 adults, along with the ratio of formal bank accounts per 1,000 adults. The data for Fintech and financial inclusion is sourced from the Global Findex Database (GFD). Furthermore, the study employs the World Governance Indicators (WGI) to evaluate institutional quality, creating a composite index by aggregating six institutional indicators: rule of law, government effectiveness, political stability, regulatory quality, control of corruption, and voice and accountability. Additional economic growth determinants are obtained from the World Bank database (World Development Indicators, WDI), including domestic credit to the private sector, government expenditure, investment, trade, school enrollment, and population growth. Detailed description of variables and statistical information are presented in Tables 1 and 2.

**Table 1:** Description of variables and data sources.

Variable	Abbreviation	Definition	Sources
No. of ATMs	Atm	Number of Automated teller machines (ATMs) (per 100,000 adults)	WDI
No. of Branches	Branches	Number of Commercial bank branches (per 100,000 adults)	WDI
No. of Accounts	Accounts	Account ownership at a financial institution or with a mobile-money-service provider (% of population ages 15+)	GFD
Made a digital payment	Mdig	The percentage of respondents who report using mobile money, a debit or credit card, or a mobile phone to make a payment from an account	GFD
Used a mobile phone or the internet to buy something online	UseMobTobuy	The percentage of respondents who report using a mobile phone or the Internet to buy something online in the past year.	GFD
Domestic credit to private sector	Priv	Domestic credit to private sector by banks refers to financial resources provided to the private sector by other depository corporations (deposit taking corporations except central banks)	WDI
GDP growth (annual %)	GDPG	Annual percentage growth rate of GDP at market prices based on constant local currency. Aggregates are based on constant 2015 prices, expressed in U.S. dollars.	WDI
General government final consumption expenditure (% of GDP)	GovExp	includes all government current expenditures for purchases of goods and services (including compensation of employees)	WDI
Gross capital formation (% of GDP)	Investment	consists of outlays on additions to the fixed assets of the economy plus net changes in the level of inventories	WDI
Inflation, GDP deflator (annual %)	Inflation	Inflation as measured by the annual growth rate of the GDP implicit deflator shows the rate of price change in the economy as a whole.	WDI
Trade (% of GDP)	Trade	Trade is the sum of exports and imports of goods and services measured as a share of gross domestic product.	WDI
School enrollment, secondary (% gross)	School	ratio of total enrollment, regardless of age, to the population of the age group that officially corresponds to the level of educat	WDI
Population growth (annual %)	PopG	Annual population growth rate for year t is the exponential rate of growth of midyear population from year t-1 to t, expressed as a percentage	WDI
institutional quality	Institutions	Aggregate of six institutional indicators: rule of law, government effectiveness, political stability, regulatory quality, control of corruption, and voice and accountability.	WGI

Note: This table presents the dependent variable and the explanatory variables that we used in the paper, their definitions the abbreviations used in empirical results, and sources of observed data. WDI stands for World Development Indicators, GFD stands for Global Findex database, WGI stands for World Governance Indicators.

**Table 2:** Descriptive statistics.

Variable	Obs	Mean	Std. Dev.	Min	Max
Atm	384	28.24	26.393	.32	117.93
Branches	393	11.845	11.7	.31	72.07
Accounts	358	41.664	23.267	.4	98.46
Mdig	264	29.528	19.652	.69	90.76
UseMobTobuy	172	10.941	12.015	.03	80.05
Priv	390	36.389	30.737	.005	177.267
GDPG	428	4.746	8.958	-50.339	153.493
GovExp	395	14.602	5.908	2.36	43.702
Investment	386	24.953	9.216	-15.917	69.603
Inflation	428	8.99	16.752	-26.7	235.515
Trade	399	74.18	35.216	4.128	305.968
School	293	70.95	27.305	5.46	141.203
PopG	432	1.647	1.455	-6.852	11.794
Institutions	432	-.605	.565	-2.273	.87

A correlation matrix was formulated to assess multicollinearity by examining the magnitude of relationships between covariates. The results, as outlined in Table 3, present a comprehensive examination of the interconnectedness among the variables, offering valuable insights into their respective associations.

**Table 3:** Matrix of correlations.

Variables	(1)	(2)	(3)	(4)	(5)	(6)	(7)	(8)	(9)	(10)	(11)	(12)	(13)	(14)
(1) Atm	1.000													
(2) Branches	0.285	1.000												
(3) Accounts	0.591	0.311	1.000											
(4) Mdig	0.493	0.220	0.870	1.000										
(5) UseMobTobuy	0.598	0.192	0.728	0.748	1.000									
(6) Priv	0.424	0.177	0.400	0.218	0.377	1.000								
(7) GDPG	0.053	0.073	0.105	0.033	0.094	-0.030	1.000							
(8) GovExp	0.218	0.123	0.158	0.192	0.190	0.025	-0.224	1.000						
(9) Investment	-0.118	0.135	0.013	-0.047	0.006	0.037	0.151	-0.078	1.000					
(10) Inflation	0.255	0.020	0.184	0.257	0.286	-0.202	0.217	0.010	-0.064	1.000				
(11) Trade	0.198	0.106	0.273	0.275	0.406	0.388	-0.206	0.307	0.250	-0.142	1.000			
(12) School	0.738	0.392	0.565	0.396	0.512	0.381	0.187	0.138	-0.087	0.295	0.062	1.000		
(13) PopG	-0.615	-0.413	-0.519	-0.354	-0.527	-0.295	-0.126	-0.140	0.077	-0.095	-0.340	-0.596	1.000	
(14) Institutions	0.451	0.247	0.613	0.424	0.412	0.357	0.131	0.105	-0.082	-0.043	0.206	0.571	-0.451	1.000

The evaluation of multicollinearity among the variables in Table 3 revealed no significant concerns. The correlation coefficients generally remained below 0.70, suggesting a low degree of interrelation among the variables. However, a noteworthy association was observed between the two Fintech variables (MDig–UseMobToBuy) and the number of formal accounts, which exceeded the 0.70 threshold. This finding indicates a strong positive correlation between having multiple formal accounts and utilizing mobile banking (USEMOBTOBUY), underscoring the increasing significance of digital financial technologies in facilitating access to financial services. Furthermore, education level (School) emerged as a vital determinant of financial inclusion, as evidenced by its robust correlations with account ownership, ATM utilization, and digital financial services (Mdig). This suggests that individuals with higher education levels are generally more financially literate and more inclined to engage with innovative financial tools. Additionally, the accessibility of ATMs plays a pivotal role in enhancing financial inclusion, as reflected in its strong associations with formal accounts and digital financial offerings.

To delve deeper into the possibility of multicollinearity, a Variance Inflation Factor (VIF) analysis was performed. The results yielded an average VIF value of 3.13 for all independent variables, significantly lower than the accepted threshold of 10. Detailed outcomes of the VIF analysis are available in Table 4.

**Table 4:** Variance Inflation Factor (VIF).

Variable	VIF	1/VIF
Accounts	8.20	0.121929
Mdig	6.98	0.143189
UseMobTobuy	3.49	0.286762
School	3.47	0.287937
Atm	3.00	0.333224
PopG	2.60	0.384475
Institutions	2.15	0.464552
Trade	2.03	0.493531
Priv	2.02	0.495700
Inflation	1.54	0.647889
Branches	1.40	0.715389
Investment	1.30	0.766469
GovExp	1.25	0.799111
**Mean VIF**	**3.03**	

The analysis reveals noteworthy positive correlations among several key variables, including Accounts, ATMs, and Mdig, based on the findings from the Pearson cross-sectional tests, reported in Table 5. This suggests that as these factors increase, they are associated with enhanced economic growth. On the other hand, our observations indicate a lack of significant correlation between other factors, such as Government Expenditure (GovExp) and Investment, and economic growth.

**Table 5:** Pre-estimation analysis results.

Variable	Pearson Cross sectional test
Atm	**71.31** ***
Branches	**14.58** ***
Accounts	**85.97** ***
Mdig	**57.39** ***
UseMobTobuy	**/**
Priv	**19.94** ***
GDPG	**5.80** ***
GovExp	**0.23**
Investment	**-0.51**
Inflation	**35.81** ***
Trade	**12.52** ***
School	16.82***
PopG	43.29***
Institutions	2.68***

***p < 0.01

**p < 0.05

*p < 0.1.

To better understand the dynamics of these relationships, we employed sophisticated statistical methods like Feasible Generalized Least Squares (FGLS) and Panel-Corrected Standard Error (PCSE) estimation techniques. These methods effectively address issues such as heteroskedasticity and cross-sectional correlations. The robustness of our results, supported by their statistical significance, underscores the reliability of these insights into the underlying drivers of economic growth in our study. By utilizing these advanced techniques, we can provide a more nuanced understanding of how various factors influence economic performance.

### 3.2 Methodology

In our analysis, we adopt the panel corrected standard error (PCSE) technique as our primary strategy for eliminating autocorrelation within the dataset. This robust approach guarantees unbiased parameter estimates and accurate standard error calculations, making it particularly effective for analyzing dynamic heterogeneous panel data characterized by temporal correlations and varying individual attributes. By integrating panel-specific and time-specific fixed effects, the PCSE method adeptly addresses unobserved heterogeneity and time-varying influences that could distort the relationships among variables, thereby significantly enhancing the reliability and validity of our model estimates. Furthermore, as a vital robustness check, we supplement the PCSE method with the feasible generalized least squares (FGLS) technique to rectify issues of heteroscedasticity and cross-sectional dependence in our panel data, which further refines the efficiency and accuracy of parameter estimates. The combination of FGLS with PCSE in our study fortifies the credibility of our empirical findings and strengthens the validity of our conclusions. To investigate the interplay between Fintech, financial inclusion, and economic growth, we examine the direct impact of Fintech and financial inclusion on economic growth by specifying a standard growth regression model as follows:

Growthit=λ+θFTit+ϕFIit+δZit+νit (1)

The regression model utilized in this study incorporates variables labeled as FT (for Fintech), FI (for financial inclusion), and Z (which consists of a series of control variables). The error term is indicated by ν, the intercept is represented by λ, and the coefficients for Fintech, financial inclusion, and the control variables are denoted by θ, ϕ, and δ, respectively. The subscripts (i) and (t) signify the specific country and the time period under analysis.

Thus, the model is structured as follows:

Growthit=λ+θFintechit+ϕFinancialinclusionit+ϕInvestmentit+ψTradeit+φGovernmentexpenditureit+λPopulationgrowthit+∫Inflationit+δschoolenrollmentit+χInstitutionsit+ϒDomesticCreditit+μit, (2)

We enhance our research methodology by integrating Fintech and financial inclusion, enabling us to thoroughly explore their complex relationship with economic growth. By including interaction terms as separate variables in our regression model, we can assess the importance of this dynamic connection. Analyzing the interaction coefficient allows us to quantitatively evaluate its influence on our research outcomes.


Growthit=λ+θFintechit+ϕFinancialinclusionit+£Investmentit+ψTradeit+φGovernmentexpenditureit+λPopulationgrowthit+∫Inflationit+δschoolenrollmentit+χInstitutionsit+ϒDomesticCreditit+¥Fintech*Financialinclusionit+μit, (3)

Our investigation centers on a rigorous analysis of Eq (3) to determine the sign and statistical significance of the interaction coefficients that elucidate the relationship between Fintech, financial inclusion, and economic growth. The complex dynamics among these variables may either be complementary or substitutive, depending on the coefficient signs. A negative coefficient indicates that Fintech is crucial for economic growth in countries with weak financial inclusion frameworks, highlighting substitutability. In contrast, a positive coefficient suggests a synergistic effect, where Fintech enhances economic growth in nations with strong financial inclusion mechanisms. As we delve into the impact of Fintech and financial inclusion on economic growth, we employ advanced methodologies such as panel corrected standard error (PCSE) and feasible generalized least squares (FGLS). Through fourteen comprehensive analyses, including seven iterations for each technique, we meticulously examine the effects of various factors on our primary outcome—economic growth. We begin by incorporating control variables along with those representing Fintech and financial inclusion, followed by the inclusion of interaction terms that capture their interdependencies. For example, (MDig*ATM, MDig*Branches, MDig*Accounts, UseMobTb*ATM, UseMobTb*Branches, and UseMobTb*Accounts) are individually introduced to delve deeper into their impact. The findings of these analyses are delineated in Tables 6 and 7 for a comprehensive evaluation.

**Table 6:** Impact of fintech, financial inclusion on economic growth in low, lower-middle, and upper-middle income countries for the period 2011, 2014, 2017, 2021: Panel-Corrected Standard Errors (PCSE) estimation. Dependent variable: Economic Growth.

	(1)	(2)	(3)	(4)	(5)	(6)	(7)	(8)	(9)	(10)	(11)	(12)	(13)
VARIABLES	PCSE	PCSE	PCSE	PCSE	PCSE	PCSE	PCSE	PCSE	PCSE	PCSE	PCSE	PCSE	PCSE
Priv	-0.0139*	-0.00075	-0.00476	-0.00486	-0.00502	-0.00305	-0.00302	0.00362	0.00531	-0.00092	-0.00191	0.00113	0.00447
	(0.00792)	(0.00948)	(0.00957)	(0.00906)	(0.00906)	(0.00938)	(0.00950)	(0.0107)	(0.0110)	(0.0104)	(0.0107)	(0.0102)	(0.00998)
GovExp	-0.0728*	-0.13***	-0.155***	-0.156***	-0.156***	-0.144***	-0.144***	-0.0772	-0.0732	-0.0992	-0.101*	-0.0860	-0.0968*
	(0.0399)	(0.0531)	(0.0531)	(0.0522)	(0.0522)	(0.0511)	(0.0511)	(0.0612)	(0.0613)	(0.0604)	(0.0605)	(0.0582)	(0.0564)
Investment	0.0668***	0.103***	0.102***	0.0980***	0.0986***	0.0978***	0.0977***	0.114**	0.114**	0.105**	0.105**	0.1000**	0.0834**
	(0.0221)	(0.0335)	(0.0331)	(0.0328)	(0.0328)	(0.0317)	(0.0318)	(0.0447)	(0.0446)	(0.0436)	(0.0436)	(0.0403)	(0.0394)
Inflation	-0.0107	-0.00864	-0.0194	-0.00441	-0.00414	-0.0116	-0.0115	0.0606	0.0642	0.0593	0.0577	0.0519	0.0555
	(0.0255)	(0.0341)	(0.0341)	(0.0342)	(0.0341)	(0.0335)	(0.0336)	(0.0427)	(0.0430)	(0.0423)	(0.0425)	(0.0418)	(0.0404)
Trade	-0.000772	-0.020**	-0.0190**	-0.0185**	-0.0184**	-0.0190**	-0.0190**	-0.032***	-0.034***	-0.027**	-0.027**	-0.029***	-0.0267**
	(0.00549)	(0.00895)	(0.00887)	(0.00892)	(0.00892)	(0.00867)	(0.00885)	(0.0114)	(0.0117)	(0.0115)	(0.0115)	(0.0110)	(0.0107)
School	0.00251	0.0104	0.0182	-0.00238	-0.00292	0.00301	0.00300	0.00518	-0.000232	-0.00101	-0.00157	-0.000391	-0.0130
	(0.0108)	(0.0149)	(0.0152)	(0.0132)	(0.0133)	(0.0127)	(0.0127)	(0.0184)	(0.0202)	(0.0170)	(0.0171)	(0.0161)	(0.0162)
PopG	0.135	-6.54e-05	-0.00561	0.0804	0.0945	0.0400	0.0400	-0.487	-0.487	-0.385	-0.361	-0.365	-0.270
	(0.180)	(0.191)	(0.189)	(0.189)	(0.192)	(0.191)	(0.191)	(0.327)	(0.326)	(0.323)	(0.330)	(0.311)	(0.302)
Institutions	1.205**	0.635	0.942	0.650	0.613	0.701	0.699	1.143	1.007	1.030	1.049	1.267	0.837
	(0.536)	(0.657)	(0.666)	(0.650)	(0.655)	(0.681)	(0.683)	(0.866)	(0.889)	(0.860)	(0.861)	(0.936)	(0.918)
Atm		-0.0181	-0.0564**					-0.0171	-0.00690				
		(0.0133)	(0.0233)					(0.0154)	(0.0219)				
Mdig		0.0208	-0.0181	0.0133	0.0192	0.0216	0.0223						
		(0.0148)	(0.0244)	(0.0142)	(0.0201)	(0.0270)	(0.0460)						
**Atm_Mdig**			**0.000967** **										
			**(0.000484)**										
Branches				0.0156	0.0297					-0.00595	0.00469		
				(0.0207)	(0.0398)					(0.0240)	(0.0376)		
**Bra_Mdig**					**-0.000343**								
					**(0.000822)**								
Accounts						-0.0104	-0.0102					-0.0126	0.0266
						(0.0278)	(0.0292)					(0.0225)	(0.0261)
**Acc_Mdig**							**-1.05e-05**						
							**(0.000532)**						
UseMobTobuy								0.0305	0.0710	0.0184	0.0298	0.0341	0.310***
								(0.0333)	(0.0702)	(0.0323)	(0.0446)	(0.0373)	(0.108)
**Atm_UseM**									**-0.000672**				
									**(0.00103)**				
**Bra_UseM**											**-0.00062**		
											**(0.00169)**		
**Acc_UseM**													**-0.00359** ***
													**(0.00132)**
Constant	4.935***	5.129***	6.290***	5.775***	5.547***	5.683***	5.668***	6.112***	6.040***	6.493***	6.384***	6.875***	5.233**
	(1.333)	(1.654)	(1.733)	(1.594)	(1.684)	(1.627)	(1.790)	(2.086)	(2.085)	(2.056)	(2.076)	(2.229)	(2.237)
Observations	253	154	154	156	156	161	161	98	98	99	99	104	104
R-squared	0.078	0.178	0.199	0.173	0.174	0.163	0.163	0.222	0.225	0.209	0.210	0.201	0.254
p-value	0.00609	0.000232	7.02e-05	0.000307	0.000547	0.000494	0.000932	0.00184	0.00271	0.00361	0.00592	0.00344	0.000208

Standard errors in parentheses

*** p<0.01

** p<0.05

* p<0.1.

**Table 7:** Impact of fintech, financial inclusion on economic growth in low, lower-middle, and upper-middle income countries for the period 2011, 2014, 2017, 2021: Feasible Generalized Least Square (FGLS) estimation. Dependent variable: Economic Growth.

	(1)	(2)	(3)	(4)	(5)	(6)	(7)	(8)	(9)	(10)	(11)	(12)	(13)
VARIABLES	FGLS	FGLS	FGLS	FGLS	FGLS	FGLS	FGLS	FGLS	FGLS	FGLS	FGLS	FGLS	FGLS
Priv	-0.00846*	-0.00729	-0.00993*	-0.0107**	-0.0104*	-0.00571	-0.00503	-0.00066	0.00422	-0.00712	-0.00646	0.000149	0.00478
	(0.00475)	(0.00589)	(0.00564)	(0.00519)	(0.00533)	(0.00526)	(0.00578)	(0.00730)	(0.00705)	(0.00745)	(0.00772)	(0.00627)	(0.00610)
GovExp	-0.095***	-0.128***	-0.153***	-0.147***	-0.157***	-0.105***	-0.103***	-0.0621	-0.0646	-0.0982**	-0.097**	-0.080***	-0.132***
	(0.0258)	(0.0370)	(0.0340)	(0.0338)	(0.0350)	(0.0290)	(0.0300)	(0.0479)	(0.0475)	(0.0448)	(0.0450)	(0.0304)	(0.0311)
Investment	0.0732***	0.0835***	0.0919***	0.0730***	0.0750***	0.0879***	0.0864***	0.113***	0.107***	0.0960***	0.100***	0.122***	0.0887***
	(0.0103)	(0.0152)	(0.0141)	(0.0143)	(0.0147)	(0.0129)	(0.0138)	(0.0184)	(0.0175)	(0.0191)	(0.0189)	(0.0175)	(0.0183)
Inflation	-0.0264	-0.0229*	-0.0229*	-0.0283**	-0.0305**	-0.0135	-0.0129	0.0553*	0.0670**	0.0457	0.0487	0.0263	0.0470*
	(0.0179)	(0.0130)	(0.0121)	(0.0143)	(0.0142)	(0.00992)	(0.0102)	(0.0293)	(0.0283)	(0.0309)	(0.0313)	(0.0284)	(0.0270)
Trade	-0.00181	-0.015***	-0.0153***	-0.014***	-0.014***	-0.018***	-0.018***	-0.02***	-0.029***	-0.026***	-0.02***	-0.031***	-0.021***
	(0.00367)	(0.00527)	(0.00435)	(0.00499)	(0.00516)	(0.00456)	(0.00459)	(0.00670)	(0.00667)	(0.00572)	(0.00589)	(0.00569)	(0.00539)
School	0.00139	0.0141*	0.0193***	-0.000678	0.00142	0.00231	0.00187	0.00956	0.000265	0.000451	0.000157	0.00967	-0.00676
	(0.00583)	(0.00735)	(0.00683)	(0.00626)	(0.00682)	(0.00563)	(0.00584)	(0.0104)	(0.0103)	(0.00906)	(0.00920)	(0.00819)	(0.00904)
PopG	0.0757	0.0255	0.0145	0.0987	0.121	0.0295	0.0266	-0.62***	-0.583***	-0.365***	-0.315**	-0.304**	-0.129
	(0.108)	(0.0777)	(0.0705)	(0.0823)	(0.0867)	(0.0755)	(0.0763)	(0.176)	(0.169)	(0.137)	(0.148)	(0.122)	(0.154)
Institutions	0.556*	0.384	0.824***	0.380	0.393	0.502	0.507	1.150***	0.798*	0.966**	1.043***	1.049***	0.577
	(0.330)	(0.308)	(0.278)	(0.261)	(0.263)	(0.309)	(0.310)	(0.417)	(0.419)	(0.409)	(0.404)	(0.393)	(0.387)
Atm		-0.0188**	-0.0504***					-0.02***	-0.00618				
		(0.00811)	(0.0130)					(0.00868)	(0.0137)				
Mdig		0.0197**	-0.00948	0.0165**	0.00925	0.0172	0.0221						
		(0.00793)	(0.00946)	(0.00727)	(0.0109)	(0.0129)	(0.0208)						
**Atm_Mdig**			**0.000792** ***										
			**(0.000268)**										
Branches				0.0164**	-0.00554					-0.00902	-0.0198		
				(0.00796)	(0.0255)					(0.0136)	(0.0231)		
**Bra_Mdig**					**0.000528**								
					**(0.000574)**								
Accounts						-0.00529	-0.00479					-0.031***	0.0185
						(0.0142)	(0.0142)					(0.0107)	(0.0153)
**Acc_Mdig**							**-6.67e-05**						
							**(0.000217)**						
UseMobTobuy								0.0328*	0.100***	0.0331	0.0202	0.0564***	0.305***
								(0.0191)	(0.0349)	(0.0216)	(0.0290)	(0.0206)	(0.0589)
**Atm_UseM**									**-0.0013** **				
									**(0.000590)**				
**Bra_UseM**											**0.00128**		
											**(0.00158)**		
**Acc_UseM**													**-0.003** ***
													**(0.000710)**
Constant	4.817***	5.115***	6.069***	5.976***	6.074***	5.400***	5.371***	6.126***	5.820***	6.777***	6.657***	6.327***	4.881***
	(0.742)	(0.790)	(0.737)	(0.686)	(0.704)	(0.675)	(0.679)	(1.186)	(1.177)	(1.144)	(1.161)	(0.955)	(1.136)
Observations	253	154	154	156	156	161	161	98	98	99	99	104	104
p-value	0.00	0.00	0.00	0.00	0.00	0.00	0.00	0.00	0.00	0.00	0.00	0.00	0.00

Standard errors in parentheses

*** p<0.01

** p<0.05

* p<0.1.

## 4. Results and discussion

The evaluation of model estimations begins with a detailed examination of the results displayed in Table 6, which utilizes annual data from our study. Our analysis primarily targets the dependent variable of economic growth (GDPG), while rigorously considering a range of independent variables, including Fintech, financial inclusion, institutional factors, government expenditure, investment levels, inflation, trade dynamics, school enrollment rates, credit to the private sector, and population growth. We deploy the Panel Corrected Standard Errors (PCSE) method alongside the Feasible Generalized Least Squares (FGLS) method for robustness checks. Table 6 robustly presents the findings from the PCSE analysis, while Table 7 clarifies the insights gained from the FGLS approach.

### 4.1. Fintech and economic growth (H1)

Our findings indicate that fintech, particularly through digital payments and mobile banking, exerts a significant and positive impact on economic growth, thereby validating our first hypothesis (H1): The introduction of fintech solutions, especially mobile banking and digital payment systems, significantly enhances economic growth in developing countries. The data presented in Tables 6 and 7 illustrate a notable and constructive effect of the fintech variables—made digital payments (MDig) and mobile online purchases (UseMobTobuy)—on economic growth in these regions. Although the PCSE estimation method does not indicate a statistically significant association, the FGLS estimation consistently reveals a significant impact at the 0.01% threshold across nearly all model configurations. Our findings align with the established body of literature regarding the role of financial innovation in promoting economic development in developing nations [31, 32, 57, 68]. This strengthens the notion that enhancements in digital financial services—especially concerning mobile payments—are in harmony with ongoing initiatives aimed at fostering economic growth and financial inclusion. By integrating fintech solutions within existing economic structures, developing countries may effectively overcome current barriers to investment and market accessibility, supporting efforts to alleviate poverty and accelerate economic advancement.

### 4.2. Financial inclusion and economic growth (H2)

The data presented in Table 6, spanning columns 1 to 13, reveal several significant insights. Our analysis indicates a detrimental association between the presence of ATMs and the number of official accounts in financial institutions with economic growth across nearly all specifications in Table 6 (PCSE) and Table 5 (FGLS). In contrast, the number of branches per 100,000 adults demonstrates a positive effect on economic growth, though this significance is limited to regression (4) in Table 7, which evaluates the integration of MDig and Branches without the interaction term. The evidence regarding the relationship between financial inclusion and economic growth appears to be mixed. While numerous studies have reported a positive link between the two [69–74], our findings imply a more nuanced relationship, highlighting a negative correlation between ATMs and formal accounts with economic growth. This finding contradicts the assertions made by Ifediora et al. [77], who identified a beneficial impact of ATMs on economic growth in Sub-Saharan Africa. Importantly, our investigation employs a more detailed approach, scrutinizing the interplay between fintech and financial inclusion. Consequently, our results challenge the validity of Hypothesis 2, which claims that financial inclusion positively and significantly influences economic growth, particularly in low- and middle-income countries. This raises important questions about the prevailing belief that broadening access to conventional financial services inherently fosters economic growth. Our research suggests that the traditional relationship between financial inclusion and economic development may be reshaped by the complex interactions between fintech innovations and financial inclusion efforts.

### 4.3. Interaction between Fintech and financial inclusion and its impact on economic growth (H3)

The regression analyses detailed in Tables 6 and 7 (and summarized in Table 8) reveal a noteworthy and positive interaction effect between the Fintech variable (digital payments) and the number of ATMs per 100,000 adults (financial inclusion) on economic growth. These findings provide robust evidence of a synergistic relationship between Fintech and financial inclusion concerning their influence on economic growth. This synergy indicates that the incorporation of technological innovations into financial services can significantly enhance the efficacy of traditional financial inclusion strategies, thereby boosting economic growth. This aligns with Fundji’s study [76] and validates our third hypothesis (H3): advancements in Fintech enhance access to financial services, subsequently driving economic growth by complementing financial inclusion efforts. Furthermore, the results support our hypothesis that the interaction and convergence of Fintech innovations with financial inclusion initiatives can alleviate the negative statistical impacts that ATMs exert on economic growth in developing nations.

**Table 8:** Summery the results of the interaction terms.

	Economic growth: GDPG (dependent variable)		
	ATM	Branches	Accounts
**MDig**	Complement	Not significant	substitute (not significant)
**UseMobTb**	Substitute	Not significant	Substitute

Additionally, the findings for the interaction terms presented in Table 7 reveal that the relationship between mobile use for online purchases (Fintech) and both the prevalence of ATMs and the count of formal accounts, which serve as indicators of financial inclusion, demonstrates a statistically significant negative correlation with economic growth. These results suggest a substitutive relationship between mobile online purchasing and traditional banking infrastructure, implying that as dependence on mobile Fintech increases, the importance of ATMs and formal accounts in fostering economic growth diminishes. This points to a potential downside in the real economy, where an excessive reliance on mobile transactions could undermine the advantages of a more holistic financial inclusion strategy, ultimately constraining broader economic growth.

This study reveals critical insights and policy implications that highlight the intricate interplay between fintech, financial inclusion, and economic growth in developing countries. Our findings demonstrate that fintech innovations—particularly in digital payments and mobile banking—substantially drive economic growth (H1), signaling their considerable potential to catalyze development in resource-limited settings. However, contrary to existing literature, we uncover a more complex relationship between financial inclusion and economic growth (H2). Our research shows mixed results, indicating that certain facets of financial inclusion, such as formal accounts and ATMs, may correlate negatively with economic growth, opposing the traditional notion that increased access to banking services automatically equates to progress.

This insight underscores that simply expanding conventional banking infrastructure will not yield the anticipated economic benefits. Moreover, our analysis of the relationship between fintech and financial inclusion (H3) reveals that these two elements are mutually reinforcing; technology-driven financial services can enhance the effectiveness of traditional inclusion strategies, suggesting that maximizing financial growth requires simultaneous investment in both domains. Conversely, the study also indicates that an overreliance on mobile transactions might undermine the influence of conventional banking systems, implying that an excessive focus on fintech solutions could inadvertently hinder a comprehensive approach to financial inclusion.

These findings advocate for balanced investment strategies that equally support traditional financial infrastructures and fintech innovations, enabling policymakers in developing nations to harness their synergistic effects on economic growth. As developing countries advance their financial sector reforms in resource-constrained environments, they must adopt a dual approach that integrates traditional banking services with fintech innovations to foster sustainable development and robust economic growth. Although a greater number of bank branches correlates positively with economic growth, the expansion of formal accounts and ATMs does not yield the same beneficial results, reinforcing the notion that mere construction of conventional infrastructure is insufficient.

Therefore, policymakers should encourage the complementary existence of fintech and traditional financial inclusion strategies, ensuring that innovations enhance rather than replace established banking systems. Achieving enhanced financial access may require a synergistic approach that combines digital services with formal accounts and incorporates fintech solutions into ATMs. Furthermore, to ensure the resilience of traditional systems and mitigate excessive dependence on mobile technology, a balanced investment in both fintech and conventional banking frameworks is vital. By acknowledging the complementary roles of fintech and traditional financial inclusion, policymakers can devise targeted strategies that promote sustainable economic growth while effectively addressing the distinct challenges confronted by their populations.

## 5. Conclusions

This study rigorously examines the intricate relationship between financial technology (Fintech), financial inclusion, and their combined effects on economic growth in developing countries. Utilizing a robust panel regression framework enhanced by Panel-Corrected Standard Errors (PCSE) and Feasible Generalized Least Squares (FGLS) techniques, the research analyzes a comprehensive dataset spanning 108 nations classified as low, lower-middle, and upper-middle income across the years 2011, 2014, 2017, and 2021. The analysis centers on vital variables related to Fintech and financial inclusion, measured through both access and utilization metrics, alongside various determinants of economic growth. Specifically, the evaluation of Fintech focuses on the percentage of individuals over 15 who engage in digital payment systems and those using mobile devices or the internet for e-commerce. In contrast, financial inclusion is quantified through access indicators, including the density of automated teller machines (ATMs) and commercial bank branches per 100,000 adults, as well as the number of formal banking accounts per 1,000 adults.

The study’s findings provide compelling evidence of the complex interplay between Fintech, financial inclusion, and economic growth in developing countries. The data substantiate the initial hypothesis that Fintech innovations serve as catalysts for economic development, revealing a positive correlation between these innovations and economic growth. Conversely, traditional measures of financial inclusion, such as ATM density and the number of formal accounts, presented an unexpected negative relationship with economic growth when analyzed alongside Fintech metrics in the same model. This challenges the widely accepted notion that increasing access to conventional financial services unequivocally fosters economic advancement and underscores the necessity of scrutinizing the interaction between Fintech and financial inclusion.

One of the pivotal conclusions from this study is the recognition that Fintech and traditional financial services are complementary. Specifically, mobile payments enhance the efficiency of existing banking infrastructures. However, the findings also indicate a significant substitutability effect, suggesting that an overreliance on mobile Fintech may undermine the relevance of traditional banking systems. This dual relationship cautions against an excessive emphasis on mobile transactions, which could potentially curtail the broader economic benefits associated with comprehensive financial inclusion strategies.

### 5.1 Policy implications

Policymakers must adopt a robust and integrated strategy that synergizes Fintech innovations with traditional banking systems, fostering meaningful partnerships between Fintech firms and established financial institutions. This approach is essential for driving sustainable economic growth and promoting financial inclusion in developing countries. Strategic investments should be prioritized in traditional banking infrastructure and mobile banking solutions, particularly in areas where positive impacts—such as mobile payments and ATM accessibility—are already evident. Furthermore, the implementation of rigorous monitoring and evaluation mechanisms is crucial to assess the cumulative impact of Fintech and conventional banking on economic outcomes, with policies being dynamically adjusted as needed. Public education campaigns should be launched to showcase the benefits of Fintech advancements and financial services, underscoring their collective potential to ignite economic growth. By executing these strategic policies, policymakers can harness the strengths of both Fintech and traditional banking, while mitigating the risks associated with the waning influence of conventional banking services.

### 5.2 Future insights

The findings of this study lay a vital foundation for a myriad of impactful future research pathways that will deepen our understanding of how Fintech, financial inclusion, and economic growth interrelate. One promising direction is to investigate the fallout from the COVID-19 pandemic on the uptake and effectiveness of Fintech solutions in promoting financial inclusion, with a keen focus on their implications in developing nations. This exploration is pivotal given the pandemic’s transformative influence on financial behaviors and access to services.

Moreover, it’s essential for future inquiries to delve into the intricate ways consumer behavior, sociocultural factors, and regulatory environments govern the adoption of Fintech and the broader concept of financial inclusion. A thorough examination of how financial literacy plays a role in shaping these dynamics could yield invaluable insights for enhancing access and usage of financial services, thereby informing policy and practice.

Furthermore, researchers must scrutinize the ramifications of Fintech innovations and financial inclusion efforts on alleviating poverty and mitigating income inequality. By conducting comparative studies that assess the differential impacts of Fintech integration across various socioeconomic strata, we can unveil the mechanisms through which digital financial services can drive inclusive growth and foster equitable economic opportunities. This multifaceted approach will not only enrich the academic discourse but also provide actionable insights for practitioners and policymakers committed to fostering a more inclusive financial landscape.
